# Expansion of CTCs from early stage lung cancer patients using a microfluidic co-culture model

**DOI:** 10.18632/oncotarget.2592

**Published:** 2014-12-01

**Authors:** Zhuo Zhang, Hiroe Shiratsuchi, Jules Lin, Guoan Chen, Rishindra M. Reddy, Ebrahim Azizi, Shamileh Fouladdel, Andrew C. Chang, Lin Lin, Hui Jiang, Meghna Waghray, Gary Luker, Diane M. Simeone, Max S. Wicha, David G. Beer, Nithya Ramnath, Sunitha Nagrath

**Affiliations:** ^1^ Department of Chemical Engineering, Department of Biomedical Engineering, University of Michigan, Ann Arbor, MI 48109; ^2^ Department of Internal Medicine, University of Michigan, Ann Arbor, MI 48109; ^3^ Department of Surgery, University of Michigan, Ann Arbor, MI 48109; ^4^ Translational Oncology Program, University of Michigan, Ann Arbor, MI 48109; ^5^ Department of Molecular and Integrative Physiology, University of Michigan, Ann Arbor, MI 48109; ^6^ Veterans Administration Ann Arbor Healthcare System, Ann Arbor, MI 48105; ^7^ Department of Biostatistics, University of Michigan, Ann Arbor, MI 48109; ^8^ Department of Radiology, Department of Biomedical Engineering, University of Michigan, Ann Arbor, MI 48109

**Keywords:** expansion of CTCs, early stage lung cancer, microfluidic co-culture

## Abstract

The potential utility of circulating tumor cells (CTCs) to guide clinical care in oncology patients has gained momentum with emerging micro- and nanotechnologies. Establishing the role of CTCs in tumor progression and metastasis depends both on enumeration and on obtaining sufficient numbers of CTCs for downstream assays. The numbers of CTCs are few in early stages of cancer, limiting detailed molecular characterization. Recent attempts in the literature to culture CTCs isolated from metastatic patients using monoculture have had limited success rates of less than 20%. Herein, we have developed a novel *in-situ* capture and culture methodology for *ex-vivo* expansion of CTCs using a three dimensional co-culture model, simulating a tumor microenvironment to support tumor development. We have successfully expanded CTCs isolated from 14 of 19 early stage lung cancer patients. Expanded lung CTCs carried mutations of the *TP53* gene identical to those observed in the matched primary tumors. Next-generation sequencing further revealed additional matched mutations between primary tumor and CTCs of cancer-related genes. This strategy sets the stage to further characterize the biology of CTCs derived from patients with early lung cancers, thereby leading to a better understanding of these putative drivers of metastasis.

## INTRODUCTION

Circulating tumor cells (CTCs) are cells shed by underlying tumors that circulate in blood and lymphatic vessels. CTCs are the likely drivers of metastasis which accounts for nearly 90% of cancer-related deaths [1]. Preventing or reducing metastases and therefore improving survival is arguably the most important goal for solid tumors, including lung cancer. Surgically resectable Stage I-III non-small cell lung cancer (NSCLC) constitutes 25% of all lung cancers, accounting for 40,000 new cases a year in the US alone [2]. Despite the performance of seemingly “curative” surgery for locally confirmed disease in these patients, more than 50% will recur in 5-years and succumb to the disease [3].

Studies indicate that circulating tumor cells (CTCs) are useful prognostic and predictive markers of recurrence and survival in patients with solid cancers, including lung cancer [4–11]. CTCs may serve as reliable biomarkers for detecting cancer recurrence earlier than other commonly used approaches, such as radiographic imaging. By the time metastasis is clinically or radiographically apparent, the tumor burden is too high for available therapies to cure the cancer. Studies in advanced lung cancer and other malignancies show that elevated numbers of CTCs are associated with reduced progression free and overall survival [12]. However, these studies also emphasize that not all CTCs lead to metastasis. It is crucial to identify the CTCs that are capable of metastasis from the ones that are mere “passengers” in order to specifically target the former. Importantly, identifying specific genetic signatures in CTCs, the earliest cells with metastasis-initiating capability, will provide new therapeutic targets. However, to achieve this, one needs to characterize CTCs from early cancers at a molecular and genomic level.

**Figure 1:**
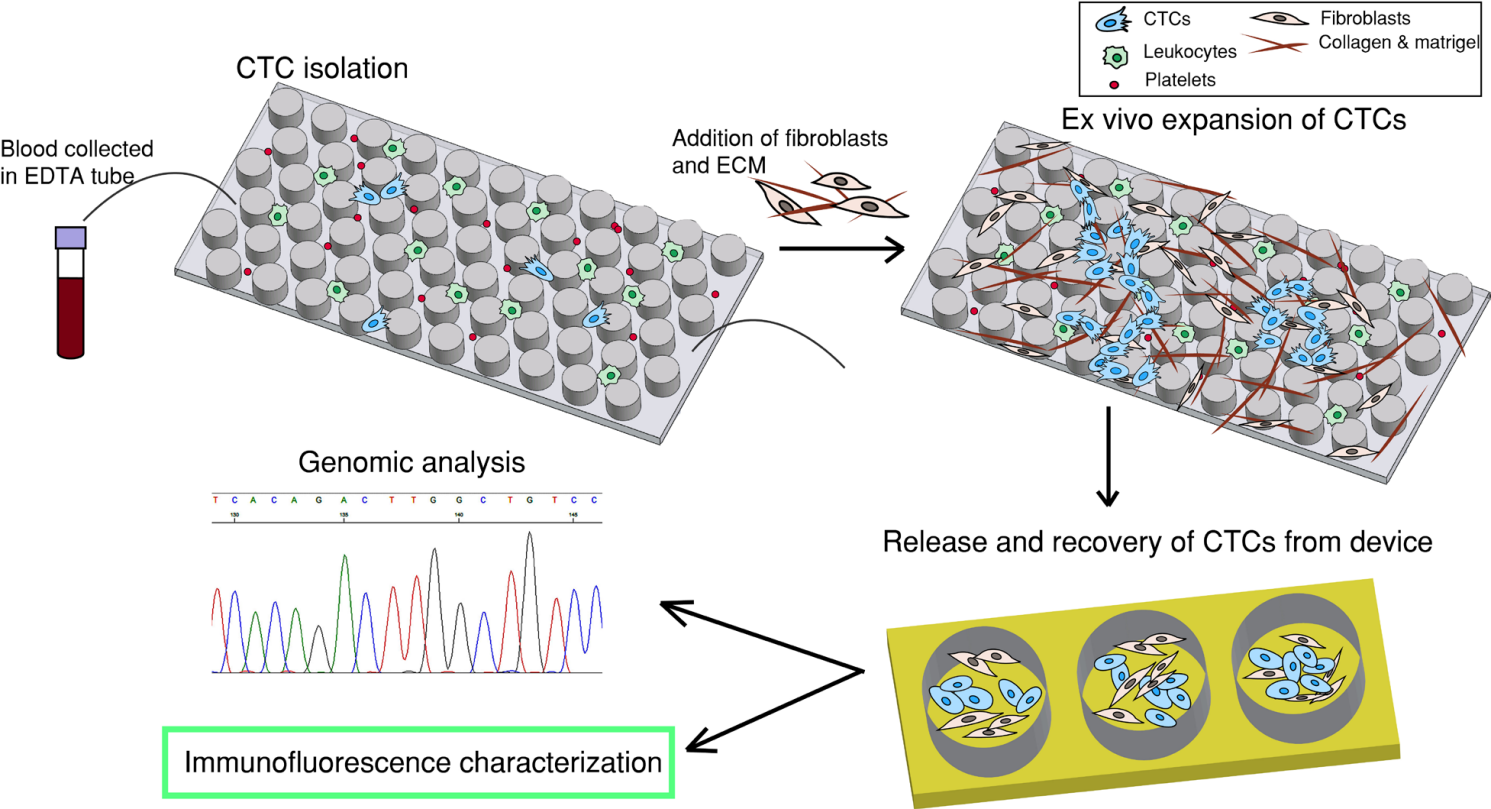
Overall strategy The first step is to capture CTCs by flowing patient blood sample through a CTC-capture chip. The second step is to introduce fibroblasts and extracellular matrix (ECM) to the same chip to establish a co-culture environment for *ex-vivo* expansion of CTCs. The third step is to release and recover CTCs from device and the fourth step is downstream characterization.

Technologies to recover rare CTCs include immunoaffinity based methods, size based filtration, dielectrophoresis, negative depletion and inertial based methods [13–24]. Among them, microfluidics offer advantages of isolating viable CTCs with relatively high yield for further analysis [25]. In the present study, we utilize an immunoaffinity-based microfluidic device and apply it to early stage lung cancer. To overcome rarity of CTCs that limit characterization for clinical utility, we isolate and further culture these CTCs on chip. To date, culturing of CTCs has only been demonstrated by a few groups, albeit in CTCs recovered from animal models or in a few patients with advanced cancers, where the likelihood of finding higher numbers of CTCs is greater [26–28]. Different from these previous approaches, and as opposed to culturing CTCs off devices, we culture captured CTCs directly on microfluidic chips. It is only after CTCs are expanded on-chip for a long period of time that they are released for subculture or downstream analysis. To facilitate CTC expansion, tumor associated fibroblasts along with extracellular matrix (ECM) proteins are introduced to construct a tumor microenvironment conducive for CTC growth (Figure 1). We herein demonstrate that rare CTCs from early stage cancers can be expanded for functional studies such as invasion and tumor spheroid forming assays, as well as sequencing of cancer related genes without pre-amplification enabling comparison between CTCs and primary tumor cells.

## RESULTS

### Characterization of CTC capture efficiency using cancer cell lines

The microfluidic CTC-capture device design is similar to the silicon based CTC-chip [21], but microfabricated with polydimethylsiloxane (PDMS) and functionalized with antibodies against epithelial cell adhesion molecule (EpCAM) as the capture agent. Although a number of variations of this technology are now available including the herringbone-chip (HB-chip) [23], we elected to work with the CTC-Chip as this platform has been well established and previously validated using lung cancer samples [9, 21]. However, the presented approach can be applied to any microfluidic chip based technologies that demonstrate high sensitivity [23, 29–31].

**Figure 2:**
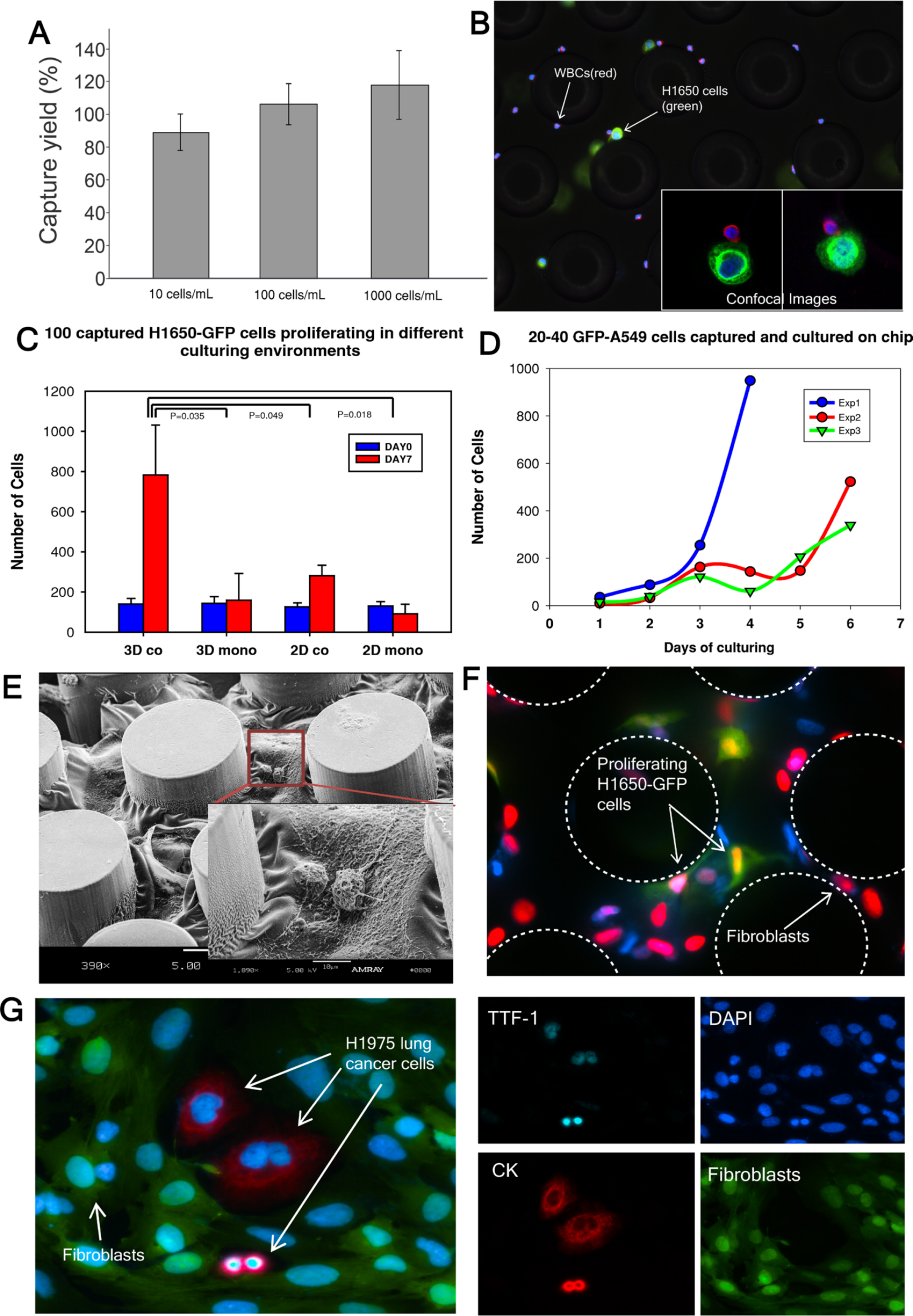
Cancer cell capture and expansion on chip **(A)** H1650 cell capture efficiency is determined (*n* = 3 for each condition). **(B)** Captured H1650 cells stained for cytokeratin 7/8 (green), CD45 (red) and DAPI (blue). **(C)** 100 H1650-GFP cells are captured and cultured in different environments (*n* = 3 for each condition). **(D)** 20–40 A549-GFP cells are captured and cultured in triplicate (indicated as Exp 1, 2 and 3). The curves characterize the growth of the cancer cells. **(E)** Scanning electron microscope (SEM) image of fibroblasts in gel cultured on chip. **(F)** EdU proliferation assay performed on cancer cells co-cultured with fibroblasts on chip. Green: cancer cells; Red: nucleus of proliferating cells; **(G)** Released H1975 lung cancer cells in a well-plate after on-chip culture. TTF-1(cyan), DAPI (blue), Cytokeratin 7/8 (red), Fibroblasts-GFP (green).

Cell capture efficiency of this device was characterized by spiking H1650 lung cancer cells at various concentrations (10,100 or 1,000 cells) into whole blood and processing the sample through the capture device. The efficiency was 87% at a 10 cells/mL concentration and almost 100% at concentrations of 100 and 1,000 cells/mL (Figure 2A). A similar experiment was performed with A549 lung cancer cells with a 60% capture rate (Figure S3). To distinguish cancer cells from blood cells for CTC enumeration, the captured H1650 lung cancer cells were immunofluorescence (IF) stained for Cytokeratin 7/8 (green), white blood cells were stained for CD45 (red) and nuclei were counterstained with DAPI (Figure 2B). The inset image demonstrates confocal images of H1650 cells captured near the posts. The image to the left is a planar image while the one to the right is a 3D z-stack image of a white blood cell adjacent to a cancer cell. These results demonstrate that our CTC-capture device can reliably isolate CTCs from whole blood with high sensitivity and efficiency even for low numbers of cells.

### Testing and optimization of *in-situ* CTC capture and culture with cancer cell lines

To determine the appropriate strategy for *in-situ* expansion of CTCs after isolation, small numbers of cancer cells (100 cells) were spiked into 1mL of blood. Subsequently, the captured cancer cells were maintained under different culture conditions and cultured up to 7 days on the chip. Four different culture environments were tested to determine optimal growing conditions for captured CTCs: (i) 3D co: cells cultured with a mix of collagen and matrigel and cancer associated fibroblasts derived from a primary pancreatic tumor; (ii) 3D mono: cells cultured only with gel; (iii) 2D co: cells cultured only with cancer associated fibroblasts and (iv) 2D mono: cells cultured without any gel nor fibroblasts. The numbers of cancer cells in the device on day 0 and day 7 were enumerated for comparison (Figure 2C). We observed that cells grown in the 3D co-culture environment exhibited the highest level of expansion, with an 8-fold (783 ± 248) increase by day 7 in culture. The 2D co-culture condition also facilitated a 3 fold (281 ± 52) cell expansion (*p* = 0.049), using t-test compared to 3D co-culture condition. This condition was less efficient than the 3D co-culture. We did not observe significant expansion using a 3D or 2D mono culture environment (3D mono: 159 ± 133, *p* = 0.035; 2D mono: 91 ± 48, *p* = 0.018). Hence, a 3D co-culture environment was selected to be the optimal condition for *in-situ* on-chip CTC expansion in our system. The growth curves of A549-GFP cells in 3D co-culture condition over the 7 day period are shown in Figure 2D. During the initial 1–4 days, the cells grew slowly, perhaps adapting to the environment; however, by day 4, the cells exhibited significant growth. Figure 2E shows a scanning electron microscope (SEM) image of fibroblasts cultured in a mix of collagen and matrigel beginning to spread in the microfluidic channel. Figure 2F demonstrates more than 90% of H1650-GFP cells are proliferating after being cultured for 7 days.

CTCs were released from the device after 7 days of on-chip culture and further cultured in well plates for 7 days. Immunofluorescence staining was performed to validate the phenotype of the expanded cells. Figure 2G shows staining of expanded H1975 lung cancer cells with CK (red) and thyroid transcription factor 1 (TTF-1) (cyan) surrounded by GFP-labeled fibroblasts. The expression of TTF-1, a lung specific marker, was preserved in H1975 cells, known to express this marker [32], in the on-chip cultured environment.

### Isolation, expansion and characterization of CTCs from patients with early stage lung cancer

**Figure 3:**
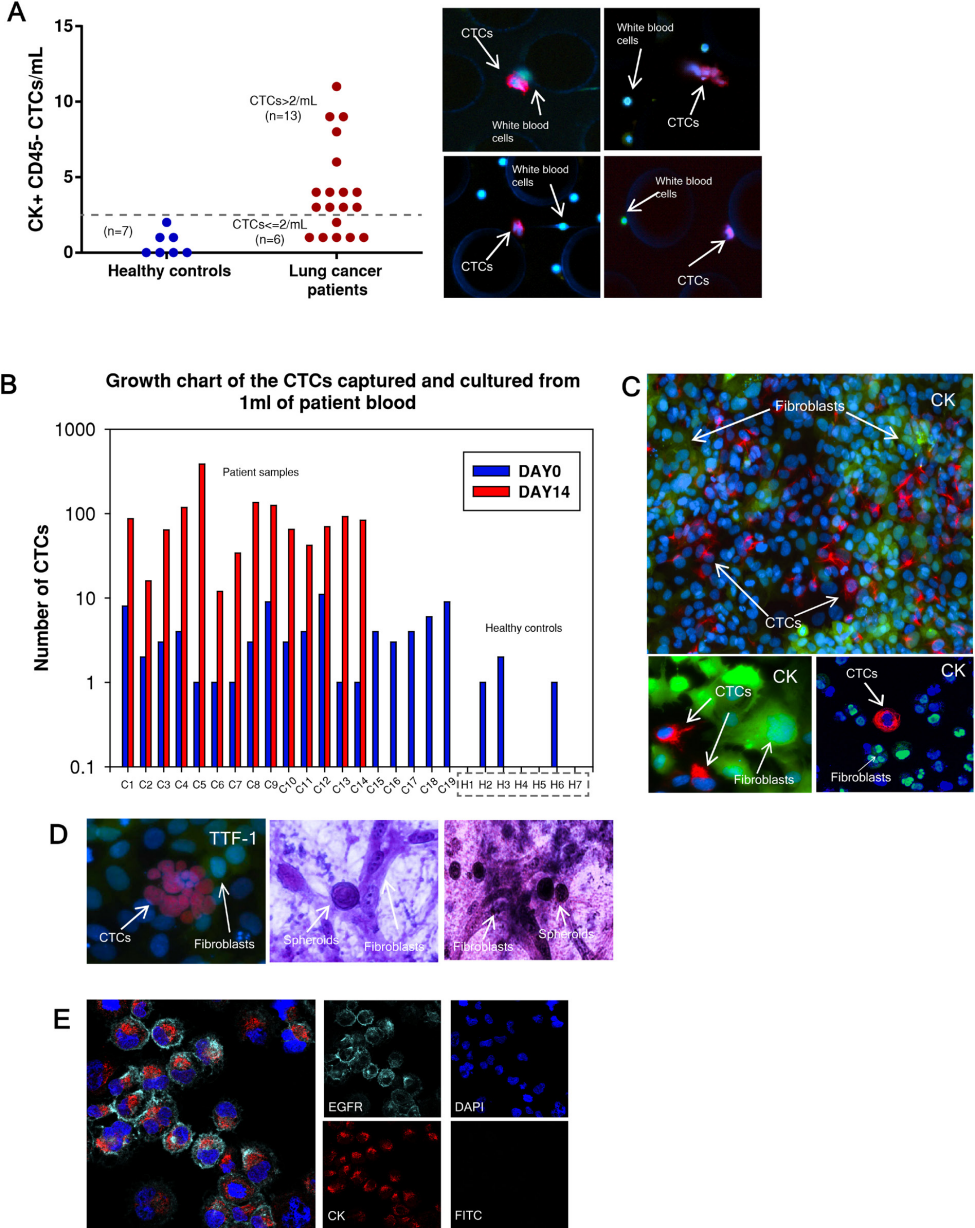
CTC capture and expansion data from patient samples **(A)** Number of CTCs captured from 1 mL of blood prior to expansion. (left) healthy controls (*n* = 7), mean = 0.6; (right) patients (*n* = 19), mean = 4. Dotted line indicates the threshold value 2 CTCs/mL. CTC positive samples are determined as >2 CTCs/mL (*n* = 13). Images to the right are patient CTCs captured in the devices. CK7/8 (red), CD45 (green). **(B)** Growth chart comparing the number of CTCs captured on day 0 (blue columns) and the number of CTCs on day 14 (red columns) after expansion. CTCs are expanded successfully from 14 out of 19 patients. **(C)** After expansion, CTCs are characterized in well-plates with CK7/8 (red) surrounded by GFP-fibroblasts. **(D)** (left) CTCs from one patient sample (C23) are stained for TTF-1 (red). (right) CTC spheroids are formed in a 3D gel assay. **(E)** CTCs sorted out from fibroblasts stained positive for EGFR (cyan) and pan-CK (red) and are negative for FITC suggesting elimination of GFP-fibroblasts.

This CTC-capture and co-culture platform confirmed with cancer cell line experiments was then utilized to test actual patient samples. Peripheral blood samples were drawn from early lung cancer patients at University of Michigan Hospital under an IRB-approved protocol. All patients involved in this study had surgically resectable early stage cancers. The blood sample from each patient was divided equally into 1–1.5 mL aliquots and run through 3–4 devices. Upon CTC isolation, one of the devices was IF stained with antibodies for enumerating CTCs on day 0. The remaining devices with cells were cultured for 7 days. Later, expanded CTCs were released and cultured up to 14 days. Nineteen patient samples were tested for capture and expansion efficiency (sample C1-C19 in Table 1). CTCs were identified in 68% of patients (total = 19, positive = 13), whereas none of the healthy volunteers (*n* = 7) showed CK positive cells greater than 2/mL (Figure 3A). These CK positive cells from healthy volunteers did not expand, demonstrate any cancer associated marker by IF or cancer associated mutation such as mutant *TP53*.

**Table 1:** Patient demographic information for samples used for quantifying expansion of CTCs

Patient	Cancer Type	Gender	Age	Tumor histology	Stage	TNM subtypes	CTC count DAY0/1ml	CTC count on DAY14
C1	lung	M	71	SCC	IA	T1bN0M0	6	87
C2	lung	M	72	SCC	IIA	T2aN1M0	2	16
C3	lung	F	50	ADC	IIB	T2bN1M0	3	64
C4	lung	M	72	ADC	IIIA	T3N1M0	4	118
C5	lung	F	74	ADC	IIA	T2bN0M0	1	386
C6	lung	M	62	ADC	IB	T2aN0M0	1	12
C7	lung	M	74	ADC	IIB	T3N0M0	1	34
C8	lung	F	79	ADC	IA	T1bN0M0	3	135
C9	lung	M	69	ADC	IIA	T2aN1M0	9	125
C10	lung	F	71	ADC	IA	T1bN0M0	3	65
C11	lung	M	86	ADC	IA	T1aN0M0	4	42
C12	lung	F	70	SCC	IIA	T2bN0M0	11	70
C13	lung	M	77	SCC	IIIA	T2aN2M0	1	92
C14	lung	M	63	SCC	IB	T2aN0M0	1	83
C15	lung	M	77	ADC	IA	T1bN0M0	4	0
C16	lung	F	74	ADC	IIA	T2bN0M0	3	0
C17	lung	F	53	SCC	IIA	T2aN1M0	4	0
C18	lung	M	93	SCC	IA	T1bN0M0	6	0
C19	lung	F	71	ADC	IA	T1bN0M0	9	0

**Figure 4:**
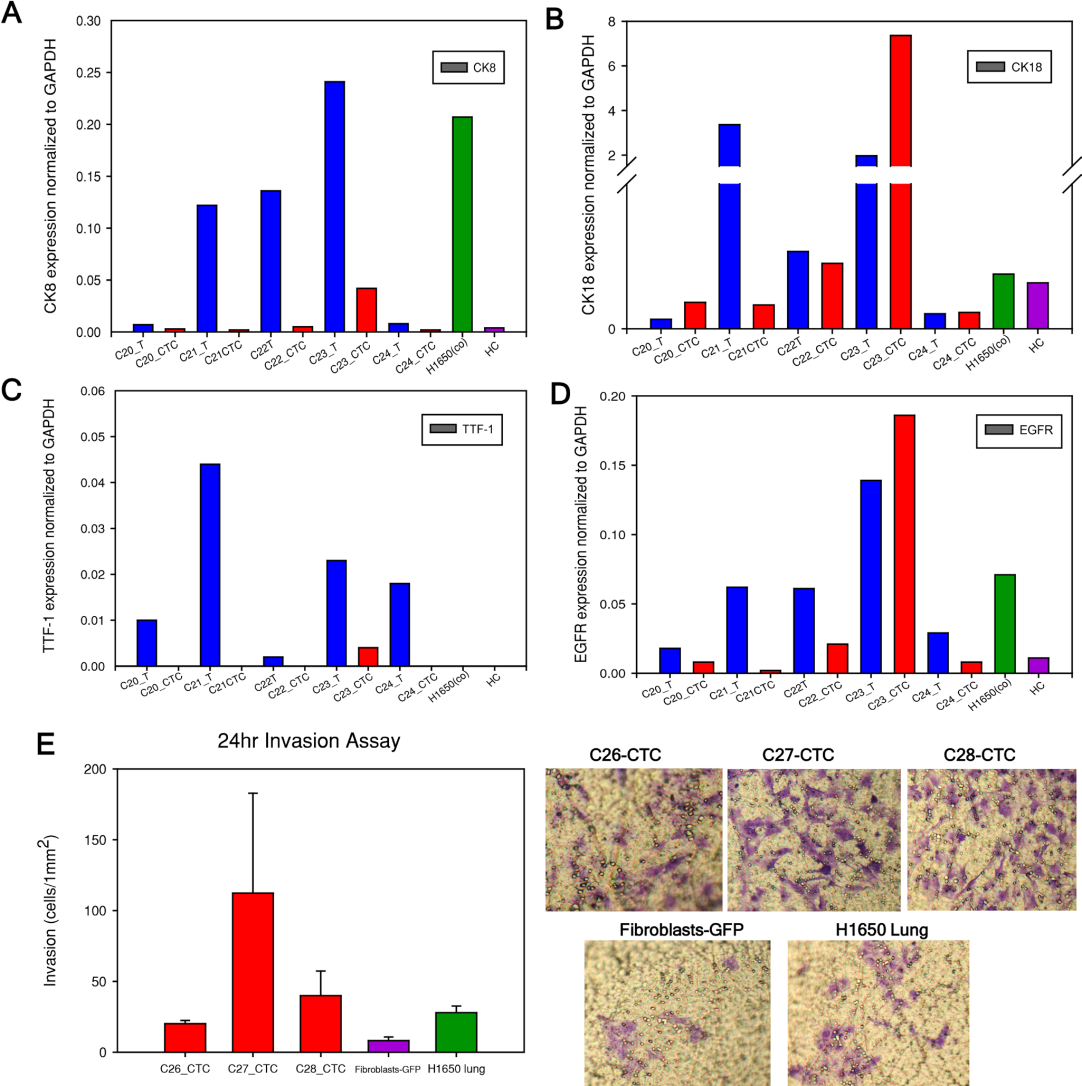
mRNA expression level in primary tumor and CTCs **(A to D)**. Cytokerain8 (*CK8*), cytokeratin18 (*CK18*), *TTF-1* and *EGFR* gene expression level normalized to *GAPDH*. Tumor and CTCs mRNA from each patient sample are examined and compared. For example, “C20_T” represents patient C20 tumor (blue column) and “C20_CTC” represents patient C20 expanded CTCs (red column). The positive control is expanded H1650-GFP cells after initially spiking in blood with 100 cells, labeled as “H1650 (co)” (green column). The negative control is one healthy control as “HC” (purple column). **(E)**. Invasion assay performed on three CTC samples, fibroblasts-GFP and H1650 cells for 24 hours. Representative images of the transwell membrane are shown on the right.

CTC numbers in these early stage patients ranged from 1–11/ml (mean = 4, median = 3, SD = 3). Patient CTCs captured on the microfluidic device on day 0 were identified as those that stained positive for CK7/8 (red) and negative for CD45 (green) (Figure 3A). Figure 3B lists 19 patient samples and 7 healthy controls with the number of CTCs captured on day 0 and on day 14 after expansion. Fourteen of 19 patient samples (73%) had successful expansion of isolated CTCs, whereas none of the CK positive cells from healthy controls demonstrated capability of expansion in culture. An average of a 54-fold increase of CTCs was observed (range 7–385 fold) in patient samples. Figure 3C depicts fluorescence images of expanded patient CTCs in well-plates stained for CK 7/8 (red) along with GFP labeled fibroblasts. In one patient (C23), the expanded CTCs were stained for TTF-1 (red) and found to be positive (Figure 3D). RT-PCR analysis further confirmed that both the primary tumor and CTCs demonstrated TTF-1 expression (Figure 4C). Additional staining for the proliferation marker Ki67 was demonstrated in the expanded CTCs as well (Figure S6B). Expanded CTCs formed spheroids in three dimension culture whereas fibroblasts did not form spheroids (Figure 3D). One CTC sample was positive for epidermal growth factor receptor (EGFR) and pan-cytokeratin (Figure 3E).

Figure 4 summarizes the normalized gene expression level of four cancer-related genes (*CK8*, *CK18*, *TTF-1* and *EGFR*). *CK8* was overexpressed in both the tumor and CTCs of sample C23 compared to healthy control (Figure 4A, Table S2). *CK18* was highly expressed in sample C22 and C23 both in tumor and CTCs (Figure 4B). *TTF-1* was expressed in both tumor and CTCs of sample C23 (Figure 4C). Importantly, *TTF-1* expression was not observed in cells from healthy control. We found higher *EGFR* expression in both tumor and CTCs from sample C22 and C23 compared with healthy control (Figure 4D). Expression of EGFR in NSCLC is associated with frequent lymph node metastasis and chemo-resistance [33]. In addition to these genes, we also characterized *β-Catenin* and *CD45* expression in tumor and CTCs (Figure S4) and found higher expression of *β-Catenin* in patient samples. *β-catenin* is involved in *WNT* signaling pathway and is important for cell survival [34]. *CD45* was negative in all CTCs samples. In addition to *GAPDH*, the expression level of all of these genes was normalized to *β-Actin* and similar patterns were observed (Figure S5). It is important to note that patient CTCs can be functionally characterized after a combined on-chip and off-chip culture strategy.

One of the key features of tumor cells that are able to leave the primary tumor environment and enter the bloodstream is a capacity to invade [35]. We therefore assessed the invasion abilities of expanded CTCs using a migration assay. All three patient CTC samples, similar to H1650 lung cancer cells, demonstrated higher invasion capacity than GFP-fibroblasts (Figure 4E). Fold increase of invaded CTCs compared to fibroblasts ranged from 1.5 to 13-fold. H1650 lung cancer cells exhibited a 2.5-fold increase compared to control fibroblasts.

### Sequencing of *TP53* gene in expanded CTCs and primary tumors of early lung patient samples

**Figure 5:**
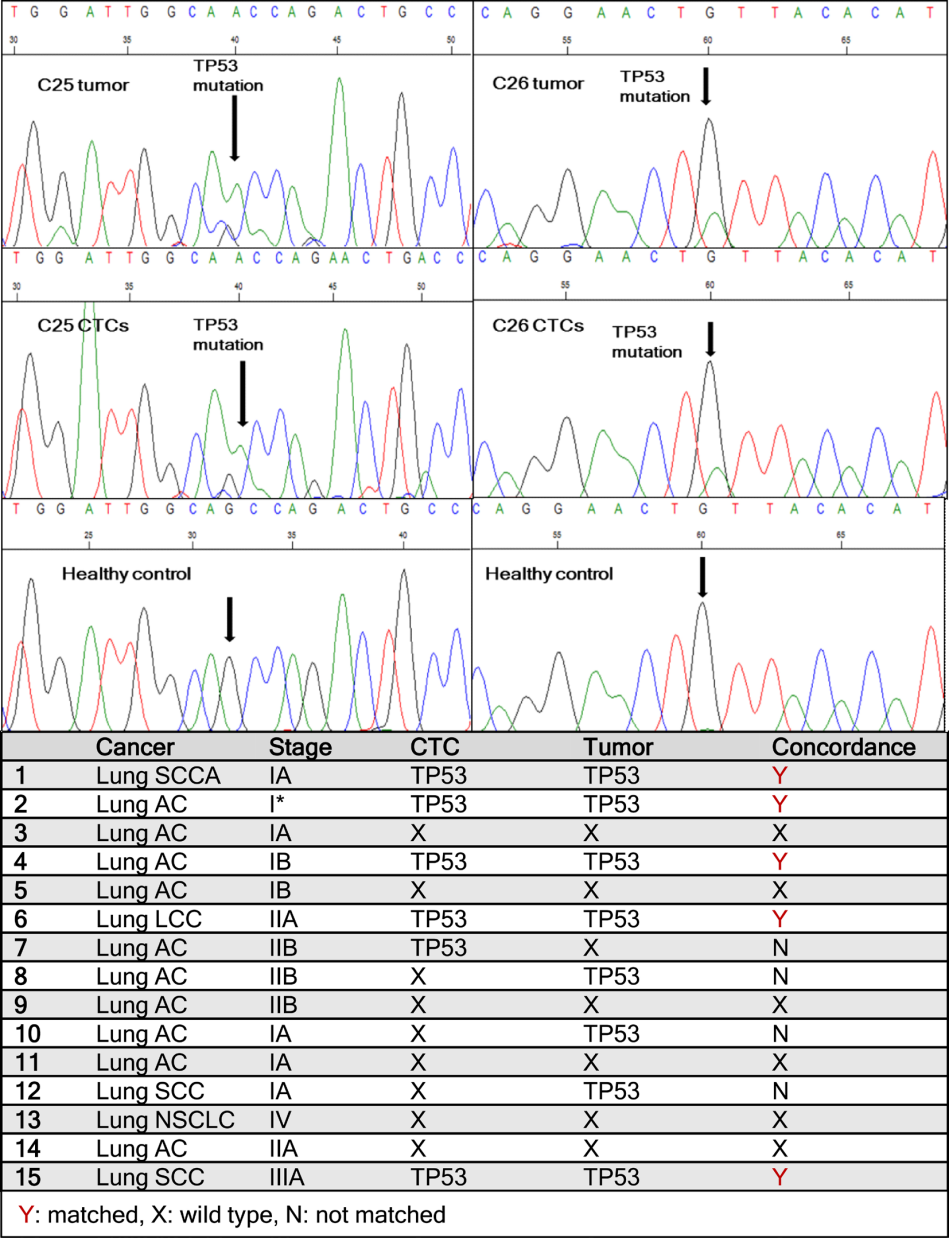
Sequencing data from patient samples One *TP53* point mutation (G to A) is found matched between primary lung tumor and cultured CTCs in patient C25. Another matched point mutation (G to A) is observed between primary tumor and CTCs in patient C26. Healthy controls showed no mutations. The table lists *TP53* mutations found in CTCs and corresponding primary tumors in all 15 lung cancer patients tested.

*TP53* is the most commonly mutated gene and is present in nearly 90% of squamous lung carcinomas and in nearly 50% of lung adenocarcinomas [36, 37]. Since mutations of *TP53* are an early event in lung tumorigenesis and believed to be preserved to maintain the malignant phenotype during tumor progression and metastatic spread [38], we hypothesized that CTCs recovered and expanded from early stage lung cancer patients should preserve *TP53* mutations present in the primary patient tumor. RNA was extracted from matching primary tumors and *ex-vivo* expanded CTCs and the tumor suppressor *TP53* gene was sequenced. Among 15 patient samples examined for the *TP53* gene, 9 (60%) had mutations in *TP53* gene. Five of the patient samples had matched mutations in primary tumors and CTCs (Figure 5, Figure S6). Figure 5 shows two matched *TP53* mutations between primary lung tumors and cultured CTCs. C25 had a G to A mutation in a non-coding region whereas C26 had G to A mutation in a coding region. However, in 3 patients, *TP53* gene mutations were noted only in the primary tumor and not the matching CTCs, whereas in one patient, the mutation was seen only in the CTCs but not in the primary tumor. The remaining patient samples analyzed exhibited wild type *TP53*. *TP53* mutations were absent from cancer associated GFP-fibroblasts as well as healthy controls, whose blood was run through the devices, co-cultured, released and processed for RNA extraction. These results demonstrate that tumor heterogeneity may exist in cells present in the primary tumor and in the circulation.

### Next-generation sequencing of expanded CTCs and primary tumors of early lung patient samples

**Figure 6:**
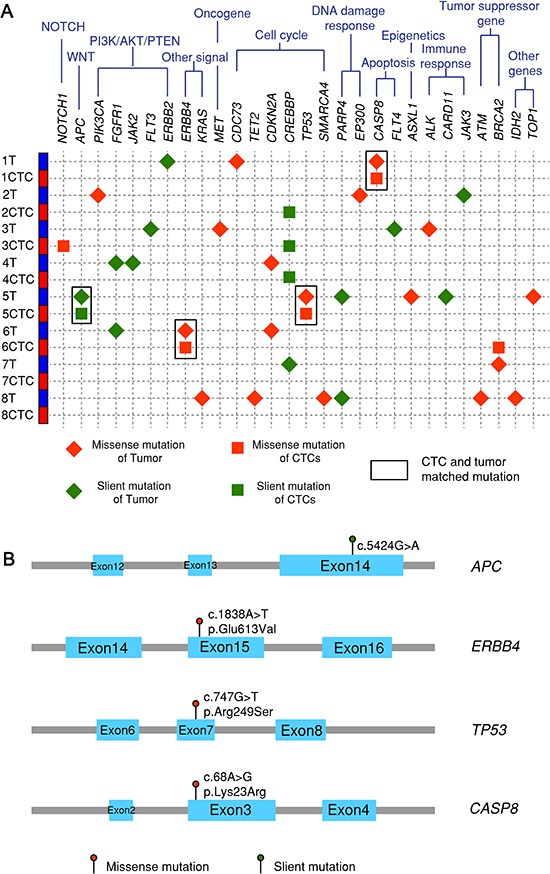
Next-generation sequencing after targeted exon enrichment **(A)** 8 paired tumor and CTC samples (C32-C39) plus one healthy control and one pure fibroblasts-GFP cell line are sequenced for 124 genes listed in the Qiagen Generead comprehensive cancer panel. Variants in each sample are identified by examining them in genome browser and confirmed by their absence in controls. **(B)** Four matched mutations are listed with their locations on exons, base change and amino acid change.

To further explore the genomic markers CTCs might carry from tumors, next-generation sequencing after targeted exon enrichment was performed with 8 paired primary tumor and CTC samples (sample C32-C39 in Table 2). Totally 124 cancer-related genes were sequenced. Figure 6A shows nonsynonymous (red) and synonymous (green) mutations with mutations in tumor in diamond shape and mutations in CTCs in square shape. Matched mutations between CTCs and primary tumor are highlighted with rectangles. Matched mutations were observed in 3 out of 8 paired CTC-tumor samples in *CASP8, APC*, *TP53* and *ERBB4* genes. The locations of the mutations on the four genes and amino acid change are shown in Figure 6B. These results demonstrate that some of the key genes involved in cancer progression are manifested in CTCs and might relate to cancer metastasis.

**Table 2:** Patient demographic information for samples used for molecular and functional characterizations

Patient	Cancer Type	Gender	Age	Tumor histology	Stage	TNM subtypes	Characterizations performed
C20	lung	F	71	ADC	IA	T1bN0M0	mRNA expression
C21	lung	F	71	ADC	IA	T1bN0M0	mRNA expression
C22	lung	M	79	SCC	IB	T2aN0M0	mRNA expression
C23	lung	F	85	ADC	IIB	T3N0M0	mRNA expression
C24	lung	M	77	SCC	IIIA	T2aN2M0	mRNA expression
C25	lung	M	68	SCC	IA	T1aN0M0	*TP53* sequencing
C26	lung	M	80	SCC	IIIA	T1aN2M0	*TP53* sequencing, Invasion assay
C27	lung	F	37	ADC	IB	T2aN0M0	Invasion assay
C28	lung	M	69	ADC	IIIB	T4N2M0	Invasion assay
C29	lung	F	72	LCC	IIA	T1aN1M0	*TP53* sequencing
C30	lung	M	80	ADC	IA	T1bNxM0	*TP53* sequencing
C31	lung	M	43	ADC	IB	T2aN0 M0	*TP53* sequencing
C32	lung	M	69	SCC	IA	T1aN0M0	Next-generation sequencing
C33	lung	M	73	ADC	IA	T1bNxM0	Next-generation sequencing
C34	lung	M	69	ADC	IIA	T2aN1M0	Next-generation sequencing
C35	lung	F	79	ADC	IA	T1bN0M0	Next-generation sequencing
C36	lung	F	72	LCC	IIA	T1aN1M0	Next-generation sequencing
C37	lung	F	74	ADC	IIA	T2bN0M0	Next-generation sequencing
C38	lung	M	42	ADC	IB	T2aN0M0	Next-generation sequencing
C39	lung	M	75	ADC	IA	T1aN0M0	Next-generation sequencing

## DISCUSSION

For over two decades, studies have shown that tumor cells shed from primary solid tumors, “CTCs” can be detected in the circulation [39, 40]. CTCs may serve as precursors to systemic metastases. Their biological and clinical significance is limited by inability to collect sufficient number of cells. Most studies to date examining biological relevance of CTCs have been carried out in animal models or patients with metastatic disease. Other efforts have been made to release CTCs using a DNA network or hydrogel but suffering cell loss and limited throughput [41, 42]. We present an *in-situ* microfluidic co-culturing model to expand captured CTCs on chip. This is achieved through creating a more physiological tumor microenvironment using a combination of collagen, matrigel along with cancer associated fibroblasts on a miniaturized device consisted of channels of only 100 μm height. This enabled better temporal and spatial control, reduction in material input, and enrichment of signaling molecules such as growth factors and cytokines [43]. This environment likely plays a key role in promoting CTC survival and expansion.

*Ex-vivo* expansion of CTCs allowed us to characterize their phenotypes in multiple aspects. CTCs, but not fibroblasts alone, were able to form spheroids in 3D gel assays (Figure 3D). Additionally, only CTCs stained positive for CK and Ki67, demonstrating that CTCs can be functionally and phenotypically distinguished from fibroblasts (Figure S7). The expanded H1975 lung cancer cells as well as patient CTCs from sample C23 expressed TTF-1 while the cultured fibroblasts lacked expression suggesting tumor specific markers are preserved in our model (Figure 2G and 3D). This observation was confirmed at the gene expression level using RT-PCR to test several cancer-related genes in primary tumors and CTCs (Figure 4). mRNA expression was heterogeneous among different patients. In some patient samples, primary tumors and CTCs demonstrated higher *CK8, CK18* and *EGFR* mRNA expression compared to healthy control. Invasion assay demonstrated that CTCs possess invasion capabilities and functional studies are feasible with expanded CTCs.

More importantly, our studies enable direct comparison of CTCs likely originating from only primary tumor (as only patients with early stage cancer with no known metastasis were chosen for this study) in early stage patients. Matched *TP53* mutations were detected in patient tumors and CTCs but absent in fibroblasts and healthy controls. This is a strong indication that mutations in *TP53* are preserved in CTCs, which may actively contribute to their ability to enable distant metastasis. We noted that 5/15 samples had matched *TP53* mutation between CTCs and primary tumor, whereas 4 samples had unmatched mutations. We believe this may reflect the inherent tumor heterogeneity observed in lung cancer as in most solid tumors [44]. For patient C26, we sequenced 4 areas of the primary tumor and noted concordance for mutations for *TP53* from 3 parts of the tumor and corresponding CTCs, suggesting that tumor heterogeneity likely plays a role in CTC shedding (Figure S8). One patient C31, with matched mutations in *TP53* recurred within 3 months in the brain and died. Another patient C5 with a 385-fold increase in CTC after expansion recurred in 3 months in the adrenal gland and died. Follow-up period in the other patients has not been long enough for recurrence data. Although a larger cohort is needed to investigate further the correlation between proliferation, mutational status of CTCs and survival, the presented data demonstrates feasibility of the approach.

Next-generation sequencing of 124 selected cancer-related genes further revealed that concordant mutations (*APC, ERBB4, CASP8*) exist in tumor and CTCs in addition to *TP53*. These genes are related to key signaling pathways for cell growth or apoptosis which collectively, might lead to tumor progression and metastasis. There are mutations unique to tumor but not in CTCs, likely due to intra-tumor heterogeneity or variable abundance of specific mutations. On the other hand, there are mutations not seen in tumor but unique to CTCs such as *NOTCH1* and *BRCA2*. Clinical significance of these mutations related to metastasis will need to be determined using larger cohorts for further investigation.

In summary, we have shown *ex-vivo* expansion of CTCs isolated from blood samples of early stage lung cancer patients, including patients with Stage I disease. We have found concordance for key genes involved in lung cancer progression using an unbiased approach (NGS). Albeit, in some cases, we found mutations in genes in the primary tumor that were not noted in the CTCs and vice-versa which raises the possibility of tumor and CTC heterogeneity. Functionally, expanded CTCs were capable of invasion compared to fibroblasts. Additionally, patients whose CTCs exhibited the greatest capacity to expand *ex-vivo* or matched TP53 mutations had earlier recurrence and died. Undoubtedly, this was an observation only in 2 patients and further studies are warranted. Finally, this microfluidic co-culture technique may open a new spectrum of opportunities for enriching early stage CTCs and aid in understanding the role of CTCs in metastasis.

## MATERIALS AND METHODS

### Device fabrication and functionalization

The CTC isolation device was designed based on the micropost architecture originally published by Nagrath et al. in 2007 (Figure S1). The device is 4.9 cm in length and 1.9 cm in width. All posts have a diameter of 100 μm. The device was made of polydimethylsiloxane (PDMS) and a 1 inch by 3 inch glass slide using standard soft lithography techniques. A SU-8 mold is made by spin coating the photoresist onto a silicon wafer and hot baking followed with exposure by UV light and developing in SU-8 developer. The PDMS device was functionalized with 4% (v/v) 3-mercaptopropyltrimethoxysilane in ethanol (Gelest), 1 μM GMBS in ethanol (Pierce), 100 μg/ml NeutrAvidin in PBS (Invitrogen) and 20 μg/ml biotinylated EpCAM in 1% BSA (R&D systems). The detailed graphic representation of this process can be found in Figure S2.

### Characterization of CTC capture efficiency with cancer cell lines

Non-small cell lung cancer cell line (H1650) cells were fluorescently-labeled with green cell tracker dye (Invitrogen). 10,100 or 1,000 labeled cells were spiked into whole blood drawn from healthy donors and flowed through the CTC capture device using a syringe pump. The number of cells captured was enumerated and capture efficiency was calculated by dividing this number by the initial cell number prior spiking. Cells in the device were counterstained with 4′,6-diamidino-2-phenylindole (DAPI) nucleus dye (Invitrogen). All experiments were conducted in triplicate.

### Optimization of CTC expansion with cancer cell lines

GFP-tagged A549 (Cell Biolab) and H1650 cells were spiked into whole blood and flowed through device. One hundred H1650-GFP cells were captured and cultured in the device. Different cell culture environments were tested and compared: 3D co-culturing: 10^5^ cancer associated fibroblasts were mixed with 0.97 mg/ml collagen I (BD Bioscience) and 50% Matrigel (BD Bioscience) [45]. The final concentration of collagen I was 0.77 mg/ml. 3D mono-culturing: only the collagen I and Matrigel mixture was added. 2D co-culturing: only fibroblasts were added. 2D mono- culturing: neither fibroblasts nor gel was added. These experiments were conducted in triplicate for each culturing condition. Collagen, Matrigel and fibroblasts were flowed into the device at a flow rate of 1 mL/hr for a total volume of 200 μl. A proliferation EdU assay (Invitrogen) was carried out to evaluate the proliferation potential of the cultured cancer cells on day 7 in device.

### Characterization of CTC capture efficiency with early lung cancer patient samples

Five mL of blood drawn from early lung cancer patients was collected in lavender EDTA tubes. Blood was flowed through the CTC-capture device at a flow rate of 1 mL/hr for 1 mL total for each device. After capture, cells were fixed with 4% paraformaldehyde and immunofluorescently stained for cytokeratin 7/8 (CK7/8) (mouse anti-human IgG2a BD bioscience) and CD45 (mouse anti-human IgG1 BD Bioscience) with Alexa Fluor 546 goat anti-mouse IgG2a and Alexa Fluor 488 goat anti-mouse IgG1 respectively. Cells were then counterstained with DAPI nucleus dye. The devices were scanned using a programmed Nikon inverted fluorescence microscope. CK7/8 and DAPI positive and CD45 negative cells were designated as CTCs and enumerated.

### CTC expansion with early lung patient samples

Peripheral blood samples were drawn from early lung cancer patients at University of Michigan Hospital under an IRB-approved protocol. Blood specimens from healthy donors were collected according to a separate IRB. One mL of blood was flowed through each CTC capturing device. Then a mixture of cancer associated fibroblasts-GFP, collagen I and Matrigel was added into the device. The device was then incubated in a 37 degree, 7.5% CO_2_ incubator for 30 minutes to facilitate gel formation. After that, media was added to the device for culturing up to 7 days. Media was RPMI complete medium (10% FBS and 1% Penicillin/Streptomycin).

### CTC release and recovery

After 7 days of on-chip culture, cells were released from the device by first incubating with collagenase for 4 hrs and then 0.25% trypsin/EDTA at 37 degree in a 7.5% CO_2_ incubator for 30 minutes. Then cells were flushed outside the device with media at 10 mL/hr flow rate for 3 mL totally. Around 90% of the cells were released from the device. The recovered population was reseeded in a well plate and cultured for an additional 7–14 days.

### Immunofluorescence cell staining

Cells in well plates were fixed with 4% PFA and permeabilized with 0.1% Triton in PBS. The cells were then blocked with 5% normal goat serum and 1% BSA in PBT solution. Cells were later immunostained with CK7/8 or TTF-1 (Santa Cruz Biotechnology) or Ki67 (Invitrogen) as well as the corresponding secondary antibodies. The number of CK7/8 positive cells was enumerated. After FACS sorting, CTCs were stained with EGFR (Cell Signaling) and pan-CK (Biolegend).

### 3D spheroid assay

CTCs together with fibroblasts cultured in well-plates were trypsinized, counted and re-suspended in a mix of collagen and Matrigel at a concentration of 10^6^ cells/mL. Two hundred μL of gel plus cell suspension was added to one well of a 48-well plate following with incubation at 37 degree and 5% CO_2_ for at least 30 min. Then 300 μL of culture medium was added to each well, which was then allowed to culture. After culturing, the plate was stained with crystal violet in methanol for imaging.

### Invasion assay

Invasion assay was performed with a 24-well transwell plate (Corning). Three CTC samples, pure fibroblasts, one H1650 lung cancer cell line were seeded at 1 × 10^5^ cells in 100 μl medium in the upper units of 8-μm-pore transwells coated with thin Matrigel. Fibroblast conditioned medium was added to the bottom well. After 24 hr incubation at 37^0^C, cells in the upper chamber were removed and invaded cells on the lower surface of the porous membrane were fixed with methanol and stained with crystal violet.

### RNA extraction

CTCs cultured in well plates and primary tumor tissue samples as well as controls underwent RNA extraction using the RNeasy Mini Kit (Qiagen). Subsequently, cDNA was made from the extracted RNA using the High Capacity cDNA Reverse Transcription Kit (Invitrogen).

### RT-PCR

RNA samples isolated from primary tumor, CTCs, positive and negative controls were first converted to cDNA and then preamplified for marker genes of interest using Cells to Ct Kit (Ambion, Life Technologies) with some modification. Finally, preamplified cDNA samples were analyzed for mRNA expression of *CK8, CK18, TTF1, EGFR, β-Catenin, CD45* (marker of WBCs) and *GAPDH* and *β-Actin* (housekeeping gene) using TaqMan probes and Gene expression kit (Applied Biosystems) on ABI 7900HT instrument (Applied Biosystems). Data were normalized to expression level of *GAPDH* or *β-Actin* and reported as fold change in expression level among different tested samples.

### *TP53* sequencing

cDNA from both primary tumors and corresponding CTC samples was PCR amplified with *TP53* primers using Expand High Fidelity PCR System (Roche). The primer sets used in this study that flanked mutated *TP53* fragments were as followings: TP53f1 5′ > 3′ GCTCCGGGGACACTTTGCGTTCG and TP53r1 5′ > 3′ GCAGCGCCTCACAACCTCCGTCAT, flanking bp 105 to 730 of *TP53* gene (of NCBI ID NM_000546), and TP53hsF 5′ > 3′ CCCCCTCCTGGCCCCTGTCATCTTC and TP53hsR 5′ > 3′ TGTTGTTGGGCAGTGCTCGCTTAGTG, which flank the mutation hot-spot region of *TP53* at bp 465 to 1136. The PCR products were characterized with gel electrophoresis and then purified using PCR Purification Kit (Qiagen). The concentration of the purified PCR products was measured and diluted for sequencing. Sequencing was performed at the University of Michigan sequencing core facility.

### Next-generation sequencing

RNA was extracted from CTCs and primary tumor and made into cDNA. Genes of interest were PCR amplified and sequenced using mixed primers from Qiagen comprehensive cancer panel for 124 cancer-related genes. Sequencing was performed with Illumina by core facility in University of Michigan. Sequencing raw data was generated initially by Qiagen online informatics platform and then analyzed by Bioinformatics core facility at University of Michigan. Called variants were filtered based on Fisher strand, allele frequency, mean read depth and mapping quality. All filterd variants were classified based on their position relative to transcript. Following classification, any variant detected in a gene not targeted by the gene panel and present in 1K Genomes data set at a frequency greater than 1% was removed. Variants appeared in healthy controls and pure fibroblasts were removed. All filtered variants were finally validated by viewing in Genome Browser for accuracy.

## SUPPLEMENTARY FIGURES


