# Supplementary material for: Expansion of CTCs from early stage lung cancer patients using a microfluidic co-culture model
Source: Oncotarget. 2014 Dec 1;5(23):12383–97. doi: 10.18632/oncotarget.2592 (PMC4323004; doi:10.18632/oncotarget.2592)
Supplement: Supplementary file 1 [file oncotarget-05-12383-s001.pdf]

SUPPLEMENTARY FIGURES

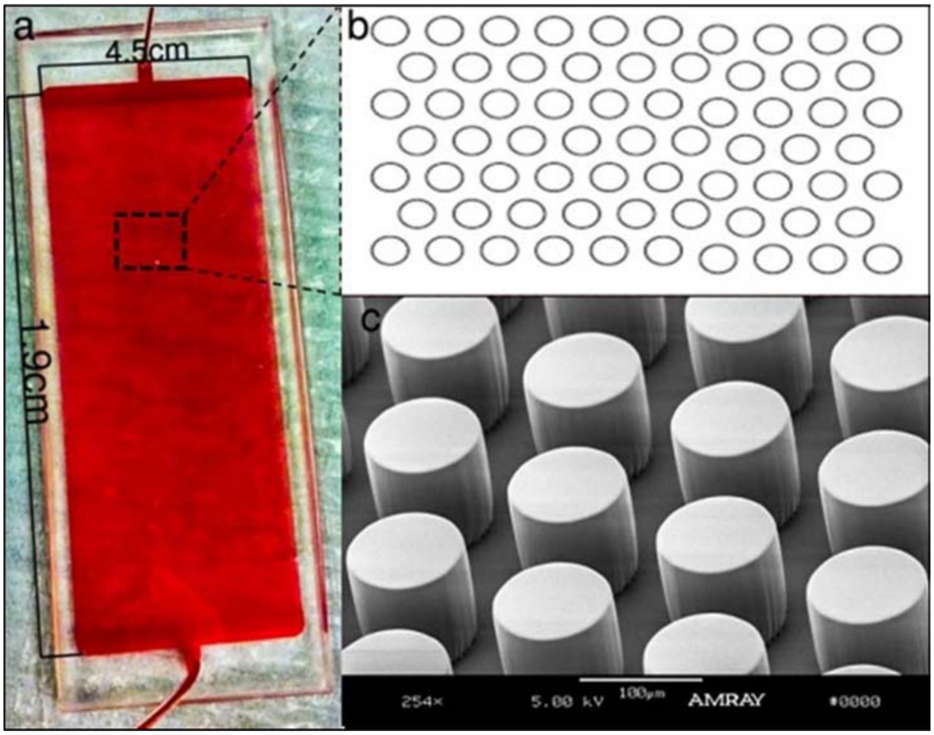

Supplementary Figure S1: CTC-capture device. (a) an actual device made of PDMS running with whole blood; **(b)** the post arrangement drawn with AutoCAD. Each post is 100 μm in diameter and the array is shifted every 10 columns; **(c)** post structures imaged with a Scanning Electron Microscope.

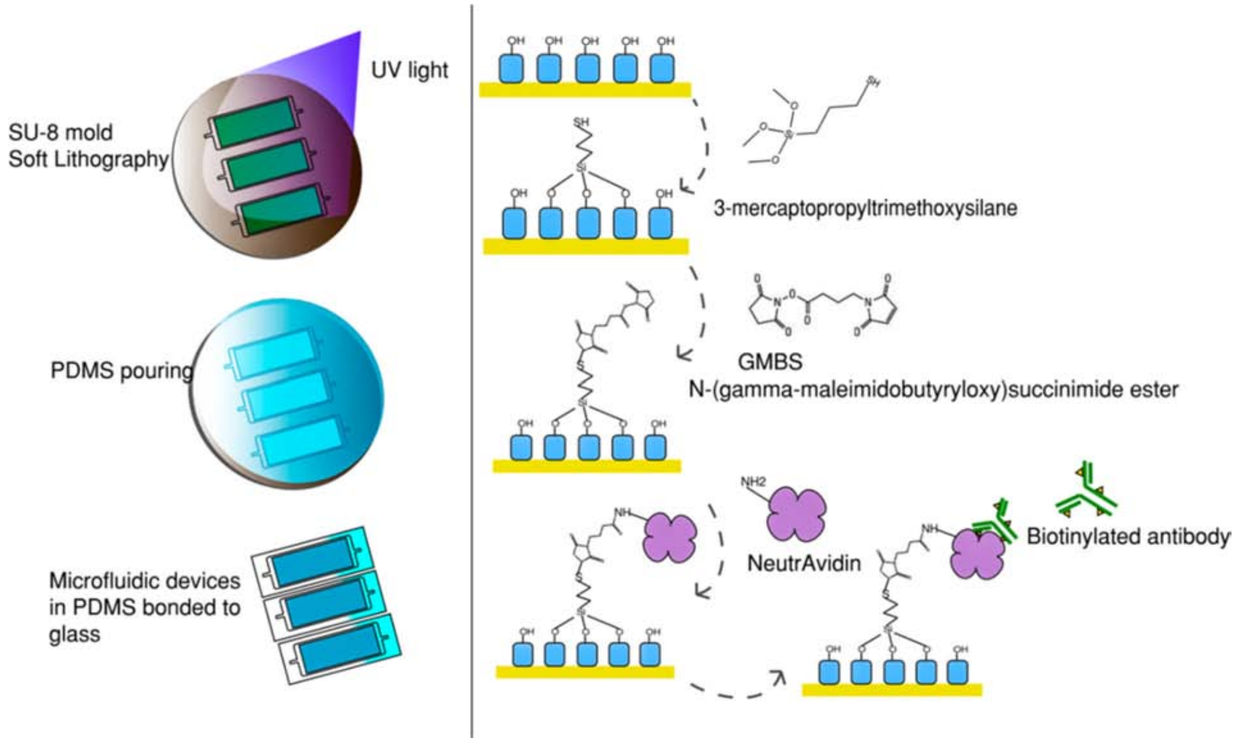

Supplementary Figure S2: Process of device fabrication and functionalization. Silane is first reacted with plasma activated glass and PDMS, then GMBS is reacted with silane and serves as a linker molecule. NeutrAvidin is then applied to link to GMBS and finally the biotinylated antibody can be attached.

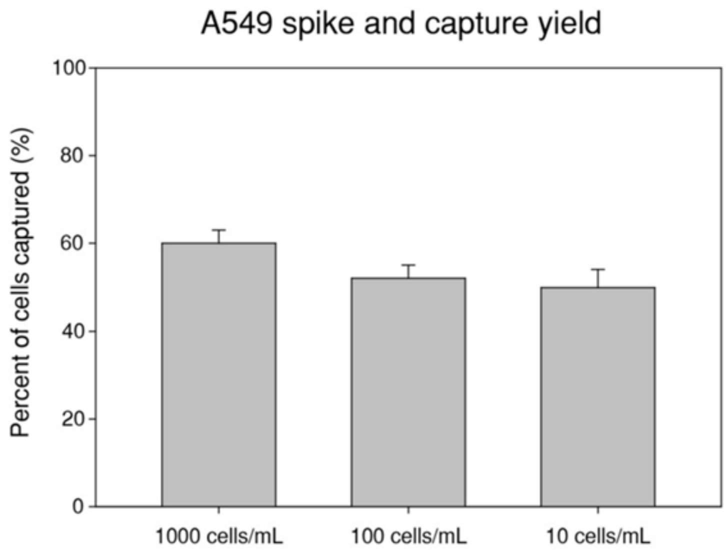

Supplementary Figure S3: A549 spike and capture from whole blood. A549 are spiked at 1000 cells, 100 cells and 10 cells in 1mL of blood. Capture efficiency is shown in the graph.

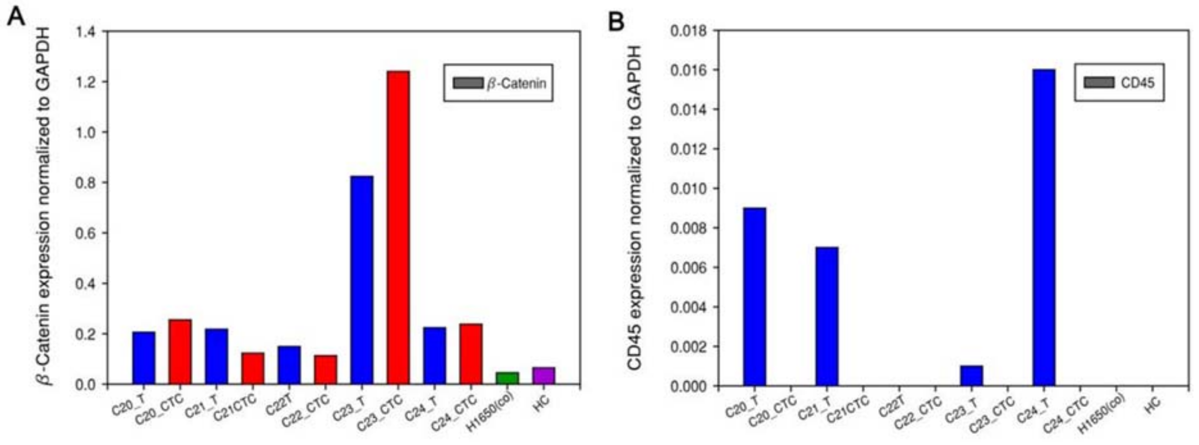

Supplementary Figure S4: ***CD45*** **and** ***β-Catenin*** **expression level in patient C20 to C24.** **(A)** *β-Catenin* is overexpressed in all patient tumors and CTCs compared to healthy control. **(B)** *CD45* is absent in all CTC samples.

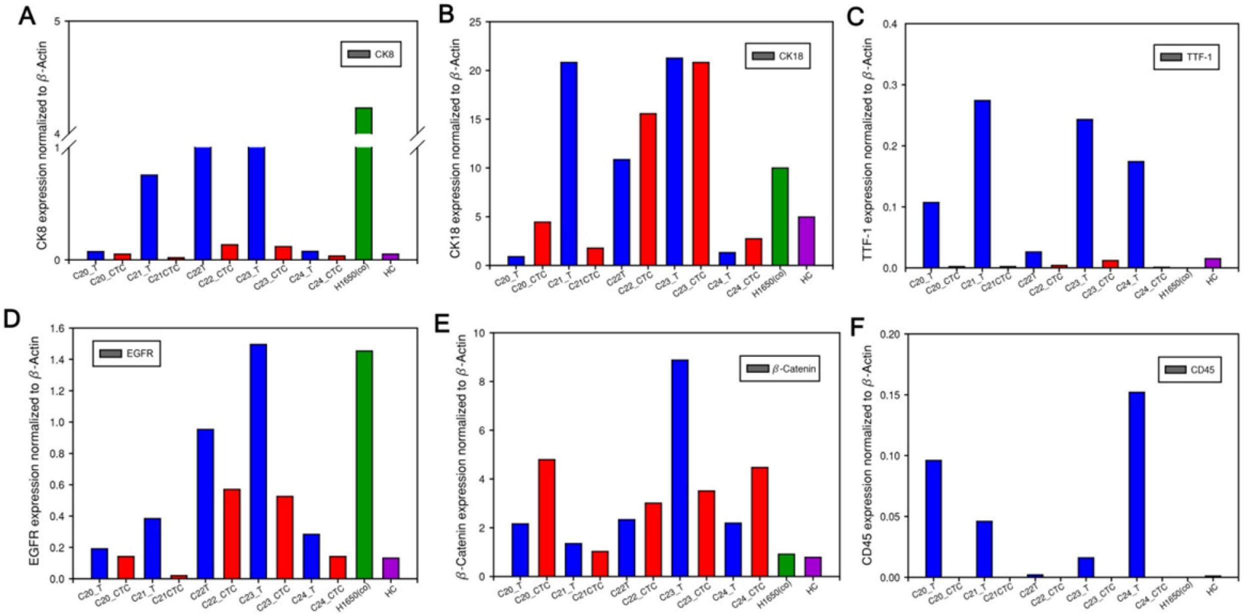

Supplementary Figure S5: mRNA expression normalized to *β-Actin* in primary tumor and CTCs. (**A** to **F**) *CK8*, *CK18*, *TTF-1*, *EGFR*, *β-Catenin* and *CD45* expression level in the same samples shown in Fig 4. Here mRNA expression is normalized to *β-Actin* while Fig 4 has mRNA expression normalized to *GAPDH*.

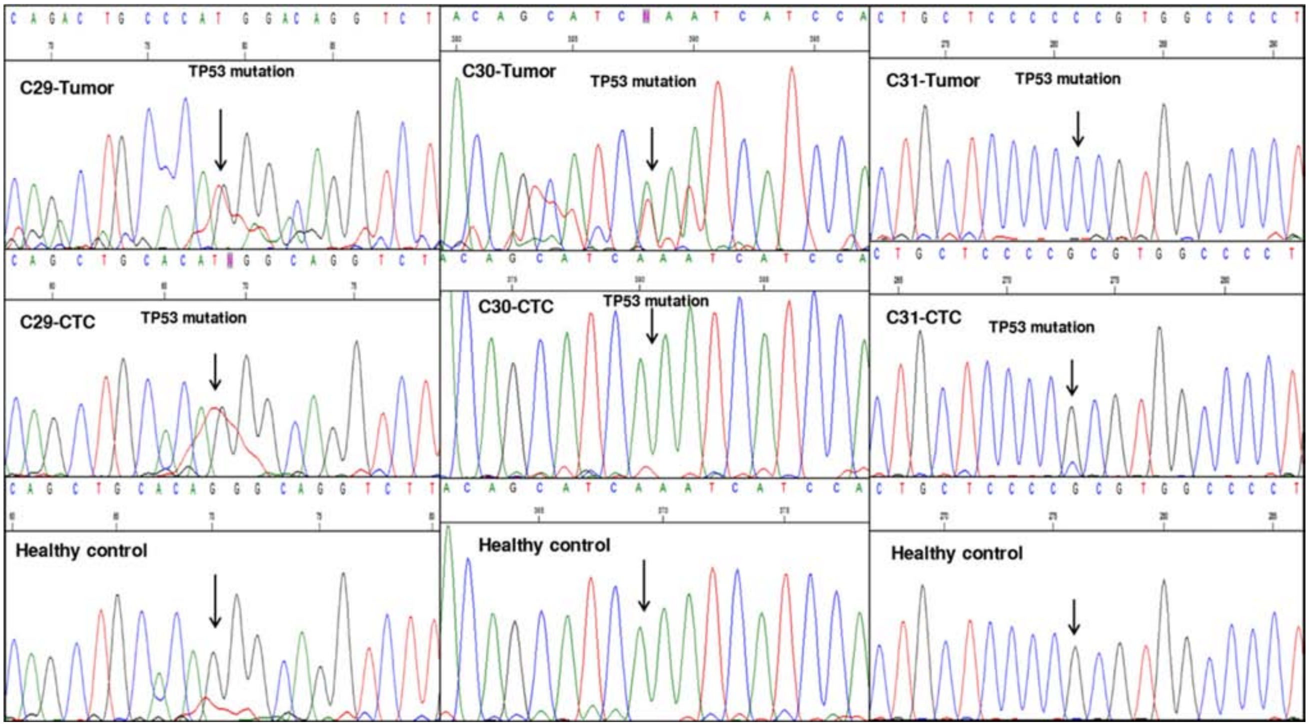

Supplementary Figure S6: Additional matched TP53 mutations between CTCs and tumors. Patient C29 has a T insertion. Patient C30 has an A to G mutation. Patient C31 has a G to C mutation.

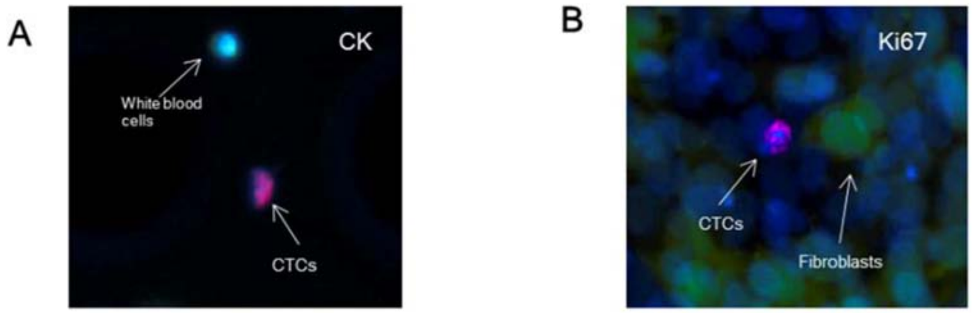

Supplementary Figure S7: Additional immunofluorescence images of CTCs. **(A)** CTCs captured on device on day 0. CTCs are stained with CK (red) and WBCs are stained with CD45 (green). **(B)** After expansion, CTCs stained positive to proliferation marker, Ki67 (purple). Fibroblasts are GFP tagged.

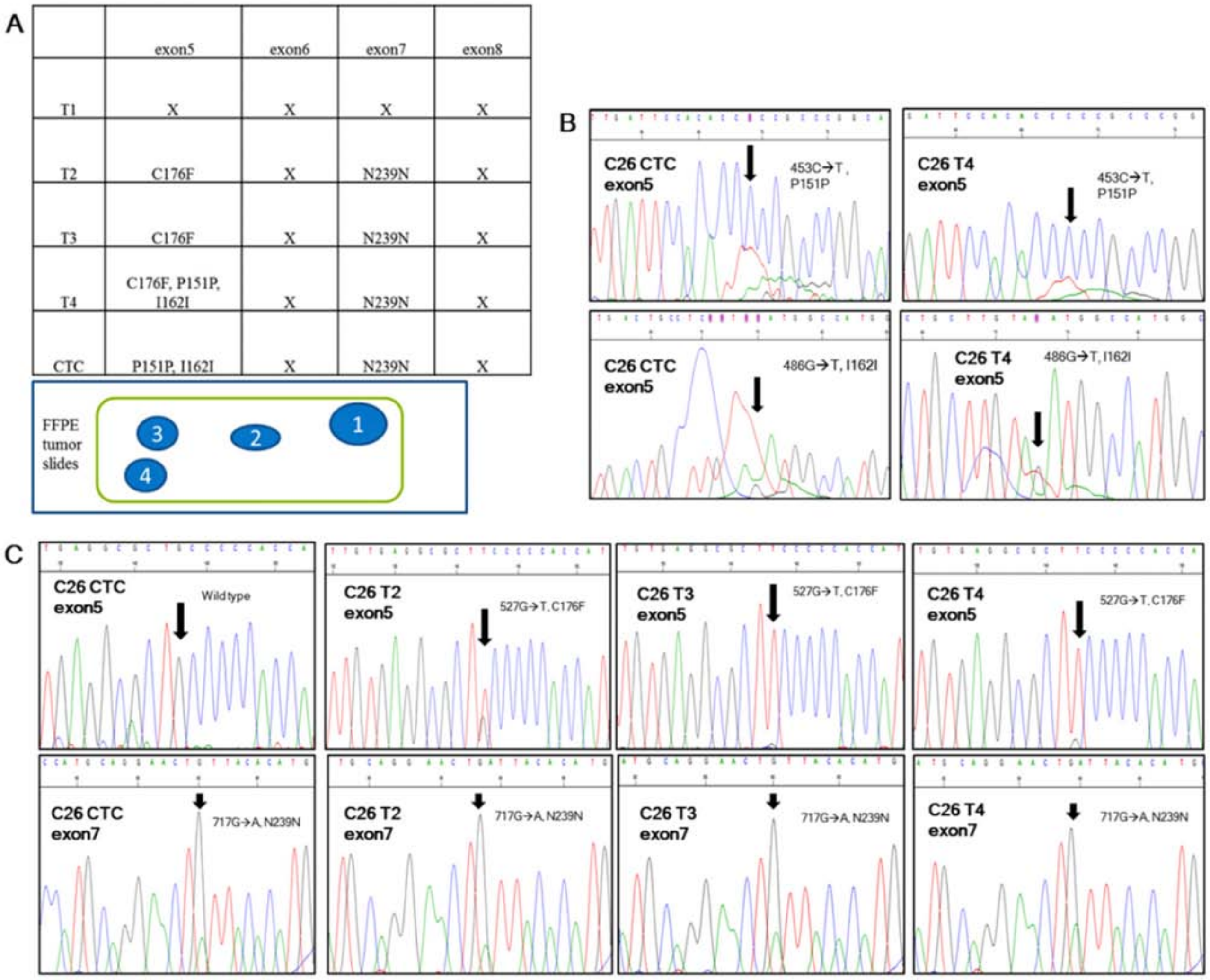

Supplementary Figure S8: *TP53* mutation status revealing tumor heterogeneity. (A) This table summarizes *TP53* mutations in CTCs and four different areas in corresponding primary tumor. “T1, 2, 3, 4” refer to tumor region1, 2, 3 or 4 from the FFPE slide shown below the table. “X”: wild-type; “C176F”: codon 176 amino acid change from C to F due to missense mutation; “P151P”: codon 151 silent mutation; “I162I”: codon 162 silent mutation; “N239N”: codon 239 silent mutation. **(B)** Chromatograms of P151P and I162I seen in CTCs and T4. **(C)** Chromatograms of C176F observed only in T2, 3 and 4 but not in CTCs, and N239N in CTCs, T2, T3 and T4.

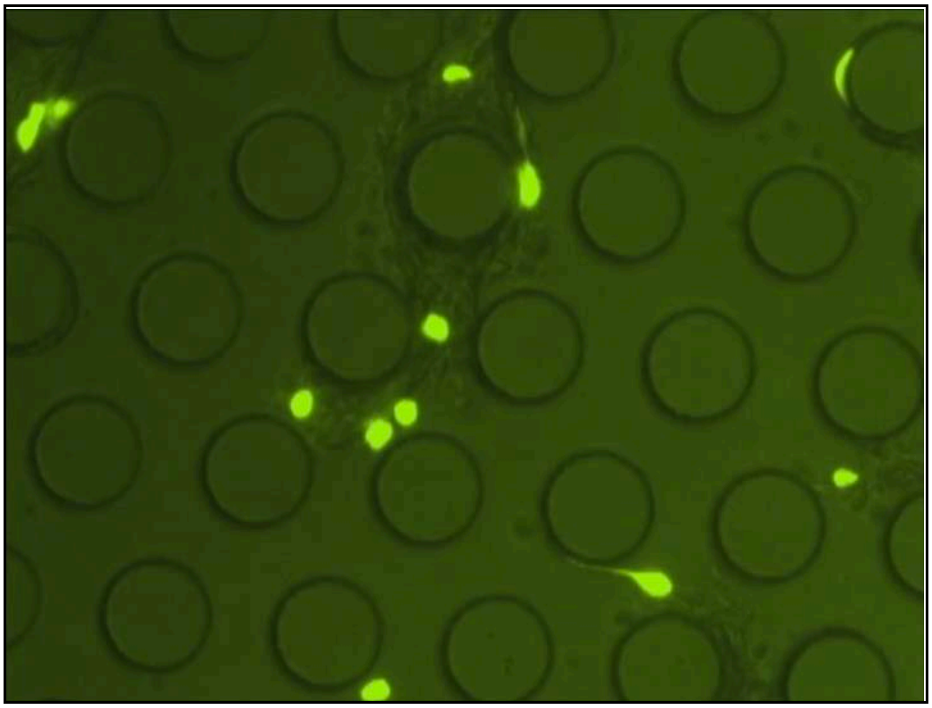

Supplementary video:
